# Dietary Polyphenols as Natural Inhibitors of α-Amylase and α-Glucosidase

**DOI:** 10.3390/life12111692

**Published:** 2022-10-25

**Authors:** Ina Ćorković, Dajana Gašo-Sokač, Anita Pichler, Josip Šimunović, Mirela Kopjar

**Affiliations:** 1 Faculty of Food Technology, Josip Juraj Strossmayer University, F. Kuhača 18, 31000 Osijek, Croatia; 2 Department of Food, Bioprocessing and Nutrition Sciences, North Carolina State University, Raleigh, NC 27695-7624, USA

**Keywords:** inhibition, enzymes, polyphenols, hyperglycemia

## Abstract

It is well known that carbohydrates are the main source of calories in most diets. However, by inhibiting carbohydrases, intake of calories is reduced and weight loss is improved. α-amylase is an enzyme that hydrolyses α-1,4 glycosidic linkages of α-linked polysaccharides, resulting in low-molecular-weight products such as glucose, maltose and maltotriose, while α-glucosidase catalyzes the hydrolysis of nonreducing α-1,4-linked glucose moieties from disaccharides or oligosaccharides. Currently, one of the most common nutritional disorders in the world is hyperglycemia. One of the new therapeutic approaches to treat this disease is the application of natural inhibitors, such as polyphenols, that control starch digestion and regulate blood glucose level. Dietary polyphenols showed potential inhibitory activity against α-amylase and α-glucosidase and this review summarizes the recently published literature that studied inhibition mechanisms and the structure–activity relationship between individual dietary polyphenols and mentioned digestive enzymes. It is known that higher binding interactions cause higher inhibitory activities; thus, different polyphenols can affect different steps in the digestion of polysaccharides. The aim of this review is to clarify these mechanisms and to introduce polyphenol-rich functional foods as potential tools for the inhibition of α-amylase and α-glucosidase.

## 1. Introduction

Currently, type II diabetes is reaching alarming rates across the world. There are different causes of this disease and are divided into modifiable risk factors, such as increased calorie intake, sedentary lifestyle and stress, and nonmodifiable risk factors, such as genetics and age. Various socioeconomic and cultural aspects cause overweight and obesity, which are consequently leading to increased risk for the disease’s development [1,2]. Type II diabetes is a result of insulin resistance and is more prevalent than type I diabetes, which is caused by insufficient insulin secretion. In addition, 5–15% of pregnant women suffer from gestational diabetes mellitus, which occurs only during pregnancy [3]. In 2021, it was reported that 537 million people were suffering from this disease, and over 90% of all diabetes cases were type II. It is predicted that this number will reach 783 million by 2045 [4]. To control type II diabetes, there are nonpharmacological approaches that include proper diet and exercise, while pharmacological approaches include drugs or insulin. Pharmacotherapy, however, can cause gastrointestinal side effects and considerable costs for patients; thus, an alternative approach is needed [5]. The use of plants containing complex substances, with different bioactivities and fewer side effects, is one of the recently proposed approaches [6]. Regulation of postprandial blood glucose level can be achieved by inhibiting α-amylase and α-glucosidase, as they are key enzymes in starch digestion [7]. Pharmaceuticals such as acarbose and voglibose are used to treat type II diabetes patients [8]. Occurrence of the side effects promoted the research of natural products that could be potent inhibitors against the enzymes and natural polyphenols that have attracted the most attention in the development of natural inhibitors [9]. Next to inhibition of carbohydrases, inhibitors of plant origin are also beneficial in weight reduction for individuals that consume great amounts of starch [10,11]. For better understanding of their health-promoting activities, it is important to determine how these plant metabolites interact with α-amylase and α-glucosidase enzymes and other dietary constituents in the human body [6]. The enzyme inhibitors are chemical compounds that reduce or completely inhibit the catalytic activity of the enzyme. The apoenzyme inhibitors can inhibit enzymes reversibly or irreversibly (permanently). The reversible apoenzyme inhibitors are of three subtypes: competitive, uncompetitive and noncompetitive or mixed-type [12]. Uncompetitive inhibitors bind to the enzyme–substrate complex at a different binding site than the substrate, while competitive inhibitors bind to the enzyme at the same binding site as the substrate. Mixed inhibitors bind to both the enzyme and enzyme–substrate complex. In the mixed or noncompetitive mechanism, conformation of the enzyme is being transferred into an inactive state and it is not able to bind the substrate or release the product, while in the competitive mechanism, the active site is reversibly blocked for the substrate molecules [13]. Definitions of terms related to enzyme inhibition are described in detail elsewhere [14].

Polyphenols are secondary plant metabolites and are introduced into the body through plant-based foods. Enzymes can react with polyphenols and these reactions are the subject of numerous recently published studies [15,16,17,18]. This review summarizes the recently published literature that have studied inhibition mechanisms and the structure–activity relationship between individual dietary polyphenols and the mentioned digestive enzymes.

## 2. Dietary Polyphenols as Antidiabetic Agents

Polyphenols commonly occur in various fruits, vegetables, spices and seeds. Their structural diversity and different health-promoting properties make them the subject of numerous studies [19,20,21,22,23]. Polyphenols are a large group of compounds ranging from simple- and low-molecular molecules, such as gallic acid, to large and complex polymers, such as condensed tannins [24,25]. They are divided into four main groups: flavonoids, phenolic acids, lignans and stilbenes. Flavonoids are the most wide-ranging group of metabolites and include flavonols, flavones, flavanones, isoflavones and anthocyanins [6]. Flavonoids are major polyphenols, with chromone moiety consisting of two rings [24,25]. Three carbon atoms of an oxygen-containing heterocyclic ring link these phenolic rings [6]. The most common flavonoids are flavonols such as quercetin and kaempferol and their glycosides, such as rutin. C-3, C-4 and C-7 positions of flavonols are usually glycosylated. Flavonols are widely distributed among fruits and vegetables and thus are prevalent in the human diet [6]. Luteolin and apigenin are flavones and they are less common in fruits and vegetables [26]. Proanthocyanidins are oligomeric flavonoids that most commonly occur in nature as oligomers of catechin and epicatechin [6]. Phenolic acids have only one phenolic ring in their structure and include two types: hydroxycinnamic and hydroxybenzoic acids and their derivates. Two phenolic rings linked with a methylene bridge form a stilbene structure. These compounds are not widely present in plants and there is often a lack of them in the human diet. However, resveratrol, as a stilbene representative, was found in grape skin, red wine and peanuts [6].

Diabetes mellitus is a multifactorial metabolic disorder in which either the concentration of the blood glucose is above 200 mg/dL or the fasting plasma glucose level is higher than 126 mg/dL [27,28]. Therapeutic strategies are based on the decrease in the post-prandial glucose levels through inhibition of degradation of the oligo- and disaccharides [29]. Polyphenols retard the absorption of glucose through inhibition of the α-amylase and α-glucosidase that are critical enzymes in the digestion of carbohydrates [30]. Drugs such as metformin, meglitinides, dipeptidyl peptidase-4 inhibitors, glucagon-like peptide-1 receptors agonists, thiazolidinediones, acarbose, miglitol and voglibose are employed to treat diabetes. Application of these drugs causes undesirable side effects, such as diarrhea, flatulence, stomach distention, weight gain and hypoglycemia and can cause serious health issues for people with liver diseases [31,32]. Abdominal discomfort, flatulence or soft stools are associated with treatments with acarbose. Passing of undigested carbohydrates from the small intestine into the colon (malabsorption) causes bacterial fermentation in the large bowel and gas production. Flatulence ranges from less than 10% to more than 50% of patients in controlled trials, which is strongly dependent on the investigational site. The nutritional habits of the patients strongly affect different incidences of gastrointestinal complaints. Enzyme activity in the distal small intestine was poor on a fiber-free diet, which caused incomplete absorption of carbohydrates in the proximal small intestine and moving into the colon. On the other hand, a fiber-rich diet caused high enzyme activity and adequate carbohydrate digestion capacity in the distal part of the small intestine [33]. Amelioration of hyperglycemia can be achieved through the application of polyphenol-rich extracts and studies have shown that polyphenols from plants are effective in humans. Thus, it is advantageous to identify the plant polyphenols that have strong inhibitory potential against α-amylase and α-glucosidase and could be used instead of acarbose to treat diabetes [34]. Inhibitors of α-amylase and α-glucosidase are divided into three groups: pseudosaccharides, proteinaceous and polyphenols. Acarbose and its derivates represent the group of pseudosaccharides [35]. Acarbose is a fermentation product of the *Actinoplanes* species that competitively inhibits α-amylase and α-glucosidase [36,37]. Structurally, it is a pseudotetrasaccharide with an unsaturated cyclitol (2,3,4-trihydroxy-5-(hydroxymethyl)-5,6-cyclohexene in a D-gluco configuration) attached to the nitrogen of 4-amino-4,6-dideoxy-D-glucopyranose, which is linked α-(1->4) to maltose. By modifying the maltose unit at the reducing end, various analogues with different inhibition properties were obtained. For example, by removing one D-glucose unit from the reducing end of acarbose, acarviosine–glucose is obtained, which inhibits yeast α-glucosidase 430 times better than acarbose. Higher inhibition activity against pancreatic α-amylase was also obtained by substituting one maltose unit with isomaltose [38].

It is important to consider the bioavailability of polyphenols when discussing their effectiveness in the prevention of diseases [39]. The pharmacokinetic and biopharmaceutical properties of polyphenols can cause a decrease in their clinical benefits as therapeutics. Affecting absorption, distribution, excretion and biotransformation, hyperglycemia disturbs the bioavailability of molecules [40]. It was reported that nanoformulations have the potential for delivering natural antidiabetic drugs such as polyphenols and different formulations are being studied to deliver these compounds to target sites [41]. Different nanoparticles ranging from 1 to 100 nm, such as liposomes, niosomes, protein-based nanoparticles, phospholipid complexes, micelles, metal nanoparticles and emulsions, are being developed, and bioavailability and controlled release of drugs can be accomplished [42]. Biodegradable, biocompatible and biofunctional biopolymers from natural sources are generally recognized as safe and are suitable to be used as carriers in different delivery systems [43,44]. By various mechanisms, nanoparticles are improving the absorption and bioavailability of polyphenols, such as the protection of the bioactive molecule from the environment of the gastrointestinal tract, prolongation of the residence time in the gut by mucoadhesion, endocytosis of the particles and/or permeabilizing effect of the polymer [45]. Factors that determine the bioavailability of polyphenols and their encapsulation using nanoparticles are described elsewhere [46].

## 3. Digestion of Carbohydrates: Role of α-Amylase and α-Glucosidase

During the digestion of foods, salivary enzymes make the first contact in the digestive systems with food components. These enzymes are mainly hydrolases that degrade carbohydrates, lipids and proteins [47]. Carbohydrates are the greatest source of calories in most diets and inhibition of their absorption can decrease calories intake and thus cause weight loss. Molecules of carbohydrates are broken down into monosaccharides by the actions of α-amylase and α-glucosidase before they are absorbed into the body [48,49]. After the digestion of carbohydrates in the mouth, the digestion process is paused in the stomach because of the acidic environmental conditions in which α-amylase is inactive. However, in the intestine, the pH is neutralized and α-amylase is secreted by the pancreas. α-glucosidase enzymes finish this breakdown into monosaccharide units as said monosaccharide units are absorbed into body [50]. Starch is a predominant ingredient of human foods and is digested by salivary and pancreatic α-amylase. The main products of its digestion are sugars, such as maltose, maltotriose, maltooligosaccharides and α-dextrin. These sugars are afterwards hydrolyzed by α-glucosidase to glucose [51]. Unlike carbohydrates in polymeric form, monosaccharides are absorbed quickly and have a high glycemic index. The glycemic index is defined as the incremental area under the blood glucose curve that occurs after the ingestion of a test food. It is expressed as a percentage of the corresponding area following an equivalent load of a reference carbohydrate (glucose or white bread). Consumption of such foods causes rapid increase in blood sugar levels and increased insulin levels. Inhibition of enzymes that digest carbohydrates may serve as an alternative to a low-glycemic-index diet [52]. Binding interactions between polyphenols and the enzymes are studied in vitro through different methods, such as IC_50_ value, which represents the concentration of an inhibitor that has exhibited 50% inhibition of enzyme activity, inhibition kinetics, molecular docking, fluorescence quenching, etc. However, it is difficult to directly determine interactions between polyphenols and enzymes since it is difficult to calculate the exact amount of enzymes reaching the small intestine and the amount of the enzymes secreted from pancreas. Other substances present in the digestive tract, such as polysaccharides and proteins, affect their interactions [53]. Main reasons for starch digestion inhibition are not only the binding interactions between polyphenols and enzymes, but also the interactions between polyphenols and starch as a result of starch microstructure alternations upon binding interactions with polyphenols [54]. Amylose as a key component of building starch intramolecular and intermolecular hydrogen-bond networks and can form different types of helical crystal structures, such as A-, B-, C- and V-types. Recently, the V-type became the subject of research of the scientific community as it showed loading capability through its helical cavity. This property enables application in food and biomedicine fields [55,56]. It was reported that polymeric proanthocyanidins can form complexes with starch, which results in a decrease in starch digestibility. V-type complexes are associated with these interactions [57].

## 4. Structure and Activity of α-Amylase

α-amylase is an enzyme present in plants, animals, bacteria and fungi [58]. It hydrolyses α-1,4-glycosidic bonds in starch, amylose, amylopectin, glycogen and other maltooligosaccharides. Glycosidic linkages are hydrolyzed in the presence of α-amylase and low-molecular-weight maltodextrins, such as maltose, maltotriose and maltotetraose, are immediately formed; this type of hydrolysis is called “multiple attack” [48,49]. This enzyme is also called α-1,4-glucan-4-glucanohydrolase and belongs to the glycosyl hydrolase family 13 of endoglycosidases [59]. Salivary and pancreatic glands produce α-amylase in the human body [60]. There are five α-amylase genes clustered in chromosome one and three of them code for salivary α-amylase, while two of them are expressed in the pancreas. Salivary and pancreatic α-amylases are composed of 496 amino acids in a single polypeptide chain [61]. Essential catalytic residues at the active site of porcine pancreatic α-amylase, determined by modelling based on the X-ray crystallographic structure, were Asp300, Asp197 and Glu223. Residues of the amino acids at the active site were in the positions where the major interactions between polyphenols and the enzyme occurred [30]. α-amylase is divided into three domains. For human pancreatic α-amylase, the first domain is the largest one, near which a bound chloride ion is found. This domain forms a central eight-stranded parallel β-barrel. The smallest domain forms a calcium binding site against the wall of the largest domain. Antiparallel β-structure makes the third domain. Human salivary α-amylase is found in glycosylated and nonglycosylated form. It also possesses a calcium binding site which is characteristic for amylases [60].

Due to polyphenols’ instability, delivery systems are often prepared to preserve them from environmental effects using different techniques. In the encapsulated form, polyphenols retain their ability to inhibit α-amylase. Table 1 presents the literature review of the inhibitory activities (expressed by IC_50_ values or the percentage of inhibition) of polyphenols against α-amylase.

## 5. Structure–Activity Relationship of Polyphenols Inhibiting α-Amylase

A study by Lo Piparo et al. [81] showed that structural features of the inhibitor are important factors determining inhibitory activity against α-amylase. Changes in the molecular structure of polyphenols, such as hydroxylation, as well as the presence of an unsaturated 2,3-bond in conjugation with a 4-carbonyl group, glycosylation, methylation, methoxylation and galloylation, also affect its inhibition activity [82,83]. In the following part, it is summarized how structural changes in certain groups of polyphenols affected inhibitory activity against α-amylase.

### 5.1. Flavonoids

#### 5.1.1. Methylation and Methoxylation of Flavonoids

Methylation and methoxylation caused reductions in the inhibitory activity and also a decrease in the hydrogen bond acceptor/donor numbers as hydrogen bonds are an important factor affecting the binding of flavonoids to α-amylase. It also caused a decrease in the polarity and improved the capacity of penetrating into the tryptophan-rich regions of proteins that are hydrophobic and are placed in the interior of the folded regions of proteins [82]. The methylation of 4′-OH on apigenin and luteolin lowered the inhibitory activity against α-amylase. The same effect was observed for the methoxylation of 7-OH on 3-methoxyapigenin and 3,7-dimethoxyapigenin [26]. However, methylation or methoxylation are effective ways to increase the bioaccessibility and bioavailability of the polyphenols in vivo [7]. Methylation or methoxylation increase the lipophilicity of flavonoids and reduced their polarity. Lipid composition of the small intestine then absorbs methylated or methoxylated flavonoids and then develop the inhibitory activity against α-amylase. It is thus necessary to take the bioavailability of the flavonoids into account when their inhibitory activity is evaluated [7].

#### 5.1.2. Hydroxylation of Flavonoids

In the study by Xiao et al. [82], it was reported that hydroxylation improved the inhibitory effects of apigenin, quercetin, kaempferol, daidzein and kushenol A, but decreased the inhibitory activity of fisetin. These findings confirm the fact that hydroxylation of flavonoids is an important factor affecting the inhibition of α-amylase [82]. Molecular hydroxylation enhanced the inhibitory activity of flavonoids as the hydroxylation at the 3′ or 3 position of flavone and the 6, 3′ or 5′ positions of flavonol and isoflavone, as well as 4′ position of flavanone, enhanced the inhibitory activity against amylase because the hydroxyl group interacts with amino acid residues at the active sites of the enzyme [7]. Among flavones (7-hydroxyflavone, chrysin, baicalein, baicalin, apigenin, luteolin, hispidulin, wogonin, tangeretin and nobiletin), the highest binding to α-amylase was observed for baicalein, which contained three hydroxyl groups on the A ring. It was concluded that the optimal number of hydroxyl groups on the A ring of flavonols is three. The addition of another hydroxyl group on ring B of flavonols decreased the affinity for α-amylase [84]. Hydroxyl groups (-OH) are important for the inhibitory activity since the inhibition depends on the formation of hydrogen bonds between sidechains of amino acids (Asp197 or Glu233) at the active site of α-amylase and -OH groups of polyphenols. Flavonoids with substitutions of -OCH_3_ at -OH in their structure are not so effective as inhibitors against α-amylase as those with -OH substituents [63].

#### 5.1.3. Glycosylation of Flavonoids

Flavonoids present in nature are often in the form of β-glycosides and mostly as the 3- and 7-O-glycosides. Quercetin has stronger inhibition activity than rutin. Monoglycoside forms (quercitrin and hyperin) of quercetin are stronger than the polyglycoside form (rutin). In addition, it was reported that luteolin had lower IC_50_ values than luteolin-7-O-β-glucoside and luteolin-4′-O-β-glucoside. These results confirmed that luteolin is a stronger inhibitor than luteolin glucoside. The same was observed for kaempferol and its glycoside forms. Increasing molecular size, polarity and nonplanar structure caused the decrease in inhibition activity. With the substitution of a hydroxyl group with glycoside, the binding of flavonoids and α-amylase was weak since steric hindrance occurred [82].

In the study by Sun and Miao [7], it was reported that 3-, 7-, 4′-monoglycosylation, diglycosylation and polyglycosylation of hydroxyl groups decreased the inhibitory activity of flavonoids and, by increasing the sugar moiety number, the inhibitory activity of the flavonoids decreased. Moreover, glycosylation substitution caused flavonoids to form nonplanar structures, which consequently decreased the binding interactions with α-amylase [7].

#### 5.1.4. Hydrogenation of the C2=C3 Double Bond of Flavonoids

The hydrogenation of the C2=C3 double bond of flavonoids decreased the binding activity for α-amylase. By comparing the inhibition activity of two flavonoids, apigenin and naringenin, it was observed that hydrogenation of the C2=C3 double bond of apigenin caused a decrease in the inhibitory effect. From these results, it can be concluded that planarity of the C ring is important for the inhibition of α-amylase, as saturated C2-C3 bonds permitted more twisting of the B ring in relation to the C ring. Near-planar structure of the molecules enabled easier entering into the hydrophobic pockets of enzymes [82]. Hydrogenation of the C2=C3 double bond transformed the near-planar structures of flavonol and flavone into a more flexible and nonplanar structure of flavanone and flavanols. It also weakened the conjugation and reduced the binding ability with α-amylase as a result of the steric hindrance [63]. The increased electron density in the C ring with C2=C3 was attributed to the binding of flavonoids with α-amylase. Molecular structures with more planarity and the C2=C3 double bond enhance the p-conjugation between rings B and C. More planar structure enables easier entering into the hydrophobic areas. The hydrogenation of the C2=C3 double bond of apigenin caused a decrease in the inhibitory activity of 21% to 5% [7].

There is a lack of consistency between the structure–affinity relationship and the structure–activity relationship of quercetin and its glycosides as α-amylase inhibitors. Hydrophobic forces cause the binding between flavonoids and α-amylase, while hydrogen bonds are not the main force that cause the binding of flavonoids to α-amylase. This occurs due to the backbone structure of flavonoids and the hydrophobic catalytic center of α-amylase. In addition, methods for determining the interactions between polyphenols and α-amylase and studying the docking model of α-amylase–polyphenol should be improved as they have a lot of drawbacks [26].

### 5.2. Catechins

Sun et al. [6] reported that galloylated catechins inhibited α-amylase more effectively than nongalloylated catechins. By the galloylation of catechins, an increase in inhibition activity was observed and the catechol-type catechins ((-)-catechin gallate and (-)-epicatechin gallate) had higher inhibition activity than the pyrogallol-type catechins (gallocatechin gallate and (-)-epigallocatechin gallate). In addition, catechins with a 3-trans structure, such as (-)-catechin gallate and gallocatechin gallate, had higher inhibitory activity than catechins with 2,3-cis structure, such as (-)-epicatechin gallate and (-)-epigallocatechin gallate [82]. It was found that trans structures caused 10 times higher inhibition activities than cis structures [26]. Three hydroxyl groups were provided by each galloyl group. These groups could potentially interact with the amino acid sidechains of α-amylase (Asp197, Glu223 and Asp300) through the formation of hydrogen bonds. In addition, the benzene ring developed hydrophobic π-π interactions (aromatic–aromatic) at the active site of the enzyme. In the galloyl group, the C=O double bonds were conjugated to the benzene ring. This caused electron delocalization and it was proposed that this led to enhanced π-π interactions with the indole ring of Trp59 of α-amylase [30]. Catechins with higher inhibitory activity had higher affinities with α-amylase, so it can be concluded that there is consistency between the binding affinity and inhibitory activity of catechins [26].

### 5.3. Proanthocyanidins and Anthocyanidins

Acidic environments are commonly favorable for most proanthocyanidins and anthocyanidins, although they are usually unstable in alkaline conditions. For determination of inhibition effects in vitro, it was proposed that a simulated digestion system be formed for anthocyanins and proanthocyanidins by using pH buffers, digestive enzymes and digestive juices. The inhibitory activity of polyphenols before and after digestion can be determined after that [7]. It is known that environmental conditions such as pH values also affect the reactions between the enzymes and polyphenols. At higher pH values, the inhibition of polyphenols increases [85].

Proanthocyanidins have polymeric structures, and, by the evaluation of α-amylase inhibition activity, it was observed that polymers have stronger inhibition activity than oligomers [82]. Dietary source and molecular structure of proanthocyanidins determined their inhibitory activity against α-amylase [7].

By studying the inhibitory activity of cyanidin and its glycosides, it was observed that cyanidin and cyanidin-3-glucoside have synergistic effects when combined with a low concentration of acarbose. Cyanidin-3-glucoside had higher inhibition activity than cyanidin and cyanidin-3-galactoside, while cyanidin-3,5-diglucoside did not show inhibition activity [82]. Cyanidin-3-glucoside had the highest inhibition activity among cyanidin-3-rutinoside, cyanidin-3,5-glucoside and peonidin-3-glucoside against α-amylase in the research conducted by Sui et al. [86]. It was observed that anthocyanins competitively inhibited the enzyme. In the study by Xu et al. [26], it was concluded that anthocyanins were not crucial for α-amylase inhibition, as yellow raspberry extracts had similar inhibitory activity against amylase as red raspberries—although yellow raspberries were rich in ellagitannins.

### 5.4. Tannins

Removing tannins from berry extracts caused a weakening in the inhibition of α-amylase [82]. In the study by Sun and Miao [7], it was reported that the inhibitory activity of tea extract ellagitannins was related to the position and occurrence of the galloyl groups in the molecule rather than to their molecular weight. It was stated that, after the consumption of berries, a synergistic effect of ellagitannins and anthocyanins on starch degradation occurs [26].

The most effective components from berry polyphenols that inhibited α-amylase were ellagitannins and proanthocyanidins. In the study by Boath et al. [87], inhibitory activity of berry polyphenols against α-amylase was studied and it was reported that synergism between polyphenol components may cause differences in the effectiveness between different berry extracts that contain proanthocyanidins and tannins but have significantly different polyphenol compositions. Tannin components, with nonspecific protein binding, prevented the enzymes from interacting with their substrates [87].

### 5.5. Hydroxycinnamic Acids and Phenolic Acids

The inhibitory effects of hydroxycinnamic and phenolic acids followed the order: caffeic acid > tannic acid > chlorogenic acid = quinic acid [82]. Chlorogenic acids from green coffee beans were evaluated for their inhibitory activity against α-amylase and the following order was obtained: dicaffeoylquinic acid > caffeoylquinic acid > caffeic acid > feruloylquinic acid > dihydrocaffeic acid > p-coumaric acid > ferulic acid > quinic acid [7]. Caffeoylquinic acids with more caffeoyl moieties had a higher inhibitory activity against α-amylase [26]. This occurred as a result of the presence of more hydroxyl groups in dicaffeoyl groups, which provided more hydroxyl groups that were important for the inhibition of α-amylase because of the formation of hydrogen bonds. In addition, dicaffeoyl molecules were more electron-rich with p-π (between double bonds and benzene) and π-π (carbonyl and double bonds) conjugated systems, which, consequently, led to stronger π-interactions with the indole ring of Trp59 [30]. Dehydroxylation and methylation of caffeic acid resulted in the formation of stable structures with a delocalized π-system with carbonyl, C=C double bonds and benzene. However, it caused a decrease in the inhibitory activity against α-amylase [30]. The esterification of gallic acid caused a reduction in the inhibitory activity. The affinities of gallic acid and its esters, termed as “gallates”, with α-amylase followed the order: gallic acid > methyl gallate > ethyl gallate > propyl gallate [84]. Hydroxybenzoic acids (salicylic and vanillic acid) barely showed any inhibitory activity, which was the opposite of the inhibitory activity of hydroxycinnamic acids against α-amylase. C=C double bonds in the molecular structure of the hydroxycinnamic acids were conjugated with the carbonyl group, which were responsible for the transfer of electrons between the benzene ring moieties and acrylic acid. Hydroxycinnamic acids formed a conjugated system, which was stabilized upon binding to the active site of α-amylase [30].

In the study by Kaeswurm et al. [88], effects of structurally diverse polyphenols on α-amylase activity were investigated. Chlorogenic acid was a representative for hydroxycinnamic acids, phlorizin for chalcones, epicatechin and epigallocatechin gallate for flavans and malvidin-3-glucoside for anthocyanins. It was observed that the inhibitor and substrate competed in their binding to the enzyme; thus, the IC_50_ values depended on the substrate concentration. Results showed that malvidin-3-glucoside and epigallocatechin gallate had significantly stronger inhibition effects than other investigated polyphenols. Epigallocatechin gallate showed a highly uncompetitive nature, malvidin showed a competitive character and phlorizin showed a weak mixed inhibition. For chlorogenic acid, pure noncompetitive inhibition was observed. It was concluded that IC_50_ values depended on the α-amylase type, specific reaction conditions and substrate concentration. Therefore, it is inconvenient to compare the obtained data with previously published literature. However, IC_50_ values could be a useful tool for the comparison of results within a measurement series, but not with other values obtained using different assays [88].

## 6. Structure and Activity of α-Glucosidase

Hydrolytic reaction and release of the α-glucose from the nonreducing ends of aryl (or alkyl)-glucosides, disaccharides or oligosaccharides is catalyzed by α-glucosidase [89]. Although they have weak activity on maltose, α-glucosidases are commonly called maltases [90]. Animals, plants and bacteria use this enzyme in various amylolytic pathways. α-glucosidase is divided into three groups according to substrate specificity. Group I are enzymes that select heterogeneous substrates such as sucrose and aryl α-glucosides. Group II and III are enzymes that select homogeneous substrates such as maltose, although enzymes in group II have high selectivity against long-chain substrates. Based on their amino acid sequences, α-glucosidases belong to the glycosyl hydrolase families 13 and 31. Enzymes in family 13 have (β/α)8-barrel-folded catalytic domain A and two additional domains labeled as B and C. Cyclodextrin glucanotransferase, α-amylase and other enzymes also belong to the glycosyl hydrolase family 13; thus, this group is subclassified into subfamilies. α-glucosidase is a member of the glucosyl hydrolase subfamily 17 [91]. More than twenty complete amino acid sequences have been reported for α-glucosidases. *Saccharomyces cerevisiae* α-glucosidase showed high similarity to *Bacillus cereus* α-glucosidase in the amino acid sequences. Conserved amino acid sequences of α-glucosidases families I and II, which were determined according to their primary structures, are clarified in the study by Chiba [92].

Two α-glucosidase isoforms are found in the small intestine. One isoform is a maltase–glucoamylase and the other is a sucrose–isomaltase. Each isoform has a different activity: the amino-terminal subunit serves as a maltase, while the carboxyl-terminal subunit acts as a glucoamylase. The maltase–glucoamylase enzyme has a higher hydrolytic activity than the sucrose–isomaltase isoform. The amino-terminal subunit of the maltase–glucoamylase isoform is labeled as the main α-glucosidase [93].

Fungi produce commercial α-glucosidases that are currently used. However, they all have limitations in the terms of slow catalytic activity, high acidic pH requirements, moderate thermostability and creation of byproducts. To improve glucose production and decrease production costs, it is important to find novel α-glucosidases. In the study by Zhai et al. [94], it was suggested that α-glucosidase be derived from environmental microbes as they showed unique physical and chemical properties, such as temperature stability, alkali tolerance and salt tolerance.

Table 2 provides a literature review of the studies that investigated the inhibitory activity of the polyphenols present in different delivery systems against the α-glucosidase enzyme. Polyphenols incorporated in delivery systems are preserved from external conditions; thus, their inhibitory activity against α-glucosidase is well preserved.

## 7. Structure–Activity Relationship of Polyphenols Inhibiting α-Glucosidase

Zhang et al. [9] reported that different approaches, such as experimental conditions, substrate properties and batches of enzymes or inhibitors, cause variations in the inhibition of enzymes. However, some important mechanisms play an essential role in the intensity of the inhibitory activity and are commonly related to the structure–activity relationship.

### 7.1. Flavonoids

Various studies have come to different conclusions regarding the inhibition of α-glucosidase by flavonoids. This occurred due to different structures of enzymes being used in the particular assays [98].

#### 7.1.1. Hydroxylation of Flavonoids

Flavones without a hydroxyl group on the 5, 6 or 7 position of the A ring did not show inhibition activity against α-glucosidase. It was concluded that 5,6,7-trihydroxylflavone structure was the decisive factor affecting inhibition of α-glucosidase. Increases in inhibitory activity could be achieved with the introduction of electron-donating or electron-withdrawing groups at position 8 of 5,6,7-trihydroxylflavone. In addition, hydroxylation on the B ring of 5,6,7-trihydroxylflavone contributed to the increase in inhibitory activity. That was proven by comparing the inhibitory activities of luteolin and apigenin, in which it was observed that hydroxylation at C-3′ of the apigenin, vitexin and isovitexin increased the inhibitory activity against α-glucosidase. The quercetin analogues with the caffeoyl structures of C-2, -3, -4 and -1′-6′ in the B/C rings of the flavonoid had higher α-glucosidase inhibition than the equivalent kaempferol derivatives. Hydroxylation of the C ring, on the other hand, caused a decrease in the inhibitory activity [99]. Genistein and daidzein are isoflavones used as traditional Chinese medicines to treat diabetes mellitus. It was reported that genistein is stronger inhibitor of yeast α-glucosidase and rat small intestine α-glucosidase than daidzein. Genistein has an additional hydroxyl group at the C5 position compared to daidzein; thus, it can be concluded that, with the formation of an extra hydrogen bond with amino acid residues, the enzyme bonding of genistein to the enzyme was stabilized [98].

#### 7.1.2. Methylation and Methoxylation of Flavonoids

The methylation on 5-OH and methoxylation on C-4′ or C-3′ flavones improved the inhibition activity. This activity was reduced by the methylation on 3′-OH of flavone. The methylation on the A ring of kurarinone and the B ring of sophoraflavanone caused a decrease in inhibition activity because of decreases in the hydrogen bond acceptor/donor numbers and the weakening of the polarity. Moreover, improvements were observed in the diffusion into the tryptophan-rich regions of proteins, which occur in the inside of the folded protein. Methoxylation at the C-3 also reduced the inhibitory activity against α-glucosidase. Flavones with 4′-methoxy groups had lower activities than 3′,4′-dimethoxyflavones. Flavones without a substitute group at the B ring also had lower inhibition activities. These results showed that inhibitory activity of 7-methoxyflavone was enhanced with the methylation of the groups at the 5′-, 3′- and 4′-positions [99].

#### 7.1.3. Glycosylation of Flavonoids

Glycosylated forms of flavonoids, such as rutin, hesperidin, hyperin, linarin, baicalin, pectolinarin and isorhamnetin 3-O-rutinoside, did not prove to be potential inhibitors of α-glucosidase. For luteolin and its glycosylated forms, the following order was observed: luteolin (monoglycoside) > luteolin-7-O-glucoside > lonicerin (polyglycoside). These results confirm that monoglycosides were stronger inhibitors of α-glucosidase than polyglycosides. The same was observed for quercetin, as this flavonoid proved to be a stronger inhibitor than isoquercetin and rutin. In blocking the enzyme, caffeoyl moiety was critical and with the substitution of the sugar moiety by a phenolic acid, α-glucosidase inhibitory activity could be enhanced [99]. The following order was observed for the inhibition of *Saccharomyces cerevisiae* α-glucosidase by flavonols: quercetin > isoquercetin = rutin, while it followed the order: isoquercetin = quercetin > rutin for *Bacillus subtilis* α-glucosidase. It was reported that flavones (luteolin, luteolin-7-O-glucoside, linarin, lonicerin, ginkgetin, isoginkgetin, bilobetin, amentoflavone, rhoifolin, baicalin and pectolinarin) inhibited α-glucosidase. Luteolin, luteolin-7-glucoside and amentoflavone had the highest inhibitory activity [98]. Increasing the molecular size and polarity and transferring to a nonplanar structure caused a decrease in the inhibitory activity. Substitution of a hydroxyl group with a glycoside caused steric hindrance to occur, which, consequently, lowered the binding between flavonoids and α-glucosidase [99].

#### 7.1.4. Hydrogenation of the C2=C3 Double Bond of Flavonoids

Weakening of the inhibitory activity occurred after hydrogenation of the C2=C3 of flavones. Apigenin showed higher inhibition activity than narigenin. It was concluded that the planarity of the C ring in flavonoids was important for binding interactions with proteins. Molecules that had saturated the C2-C3 bonds had more twisting of the B ring in relation to C ring. Near-planar structures enabled easier entering into the hydrophobic pockets in enzymes [99].

### 7.2. Catechins

For theaflavins, it was observed that the galloylation improved the inhibition activity. Their activity was closely associated with the presence of a free hydroxyl group at the 3′-position of the theaflavins [99]. *Saccharomyces cerevisiae* α-glucosidase has a hydrophobic active center. However, by increasing the hydrophilicity, the affinity for this center would not increase. The IC_50_ value of polycondensate of catechin with glyoxylic acid decreased compared to catechin. Polycondensate of catechin with glyoxylic acid could interact with amino acid residues outside of the main pocket. In this way, it could effectively hinder substrate access as a result of its large size [98].

### 7.3. Proanthocyanidins and Anthocyanidins

By studying the inhibitory activity of proanthocyanidins and anthocyanins, it was observed that oligomers had higher inhibitory activity than polymers [99]. A study on α-glucosidase inhibition by berry anthocyanins in vitro showed that acylated anthocyanins had higher inhibition activity against this enzyme than deacylated forms, which was probably a result of the stability of acylated anthocyanins at the intestinal pH [100]. Among cyanidin, cyanidin-3,5-diglucoside and cyanidin-3-galactoside, the highest inhibition against α-glucosidase was observed for cyanidin-3-galactoside. Glycosylation of anthocyanins caused an increase as the inhibition of anthocyanins followed the following order: cyanidin-3-sambubioside > cyanidin-3-glucoside > cyanidin, while methylation and methoxylation caused its decrease [99]. In the study by McDougall et al. [101], inhibition activities of blueberries, blackcurrants, raspberries and red cabbage against α-amylase and α-glucosidase were studied. It was observed that polyphenols inhibited α-amylase and α-glucosidase conversely, i.e., strawberries and raspberries were more effective against α-amylase than blueberries, blackcurrants and red cabbage, while blueberry and blackcurrant extracts that were rich in anthocyanins were more effective in inhibiting α-glucosidase. High amounts of soluble tannins in strawberry and raspberry extracts caused effective inhibition of α-amylase. Kim et al. [102] investigated the inhibitory activity of apigeninidin, luteolinidin, cyanidin, delphinidin, kuromanin, cyanidin-3-O-β-sophoroside, delpihinidin-3-O-β-glucoside and peturidin-3-O-glucoside against α-glucosidase. The obtained results showed that, among the tested compounds, delphinidin and petunidin-3-O-glucoside had inhibitory effects higher than 50% at a concentration of 100 µM.

### 7.4. Stilbenes

The glycosylation of stilbenes weakened the inhibitory activity as trans-resveratrol had higher inhibitory activity than rumexoid and glucosylated stilbene piceid. The hydroxylation of the B ring of stilbenes also caused a decrease in the inhibitory activity against α-glucosidase [99].

### 7.5. Hydroxycinnamic Acids

It was observed that chlorogenic acid (3-O-caffeoylquinic acid) and 5-O-caffeoylquinic acid, which is its structural isomer, strongly inhibited α-glucosidase, while insignificant inhibitory activity was observed for gallic acid, ferulic acid, m-hydroxycinnamic acid and p-hydroxycinnamic acid. The increase in the length of the alkyl chains caused an increase in the inhibitory activity of mono- and diketal derivates of chlorogenic acid up to the 5-nonanone derivates in the monoketals and diketals, while further increases in the alkyl chain length did not cause changes in the inhibition potency [99].

### 7.6. Tannins

Inhibition mechanisms of tannins against α-glucosidase were similar to synthetic inhibitors such as acarbose and voglibose, which are used therapeutically to control noninsulin-dependent diabetes mellitus. Tannic acid and tannin-rich compounds of red wine caused reductions in the glucose levels after consumption of meals rich in starch [6].

### 7.7. Chalcones

Chalcones are precursors of flavonoids and isoflavonoids. These compounds showed potent inhibitory activity against α-glucosidase. Aminochalcones showed strong inhibition activity against α-glucosidase, while nonaminochalcones showed a lack of this property. The monoglycosylation of iriflophenone caused a decrease in the inhibition potency [99].

**Figure 1:**
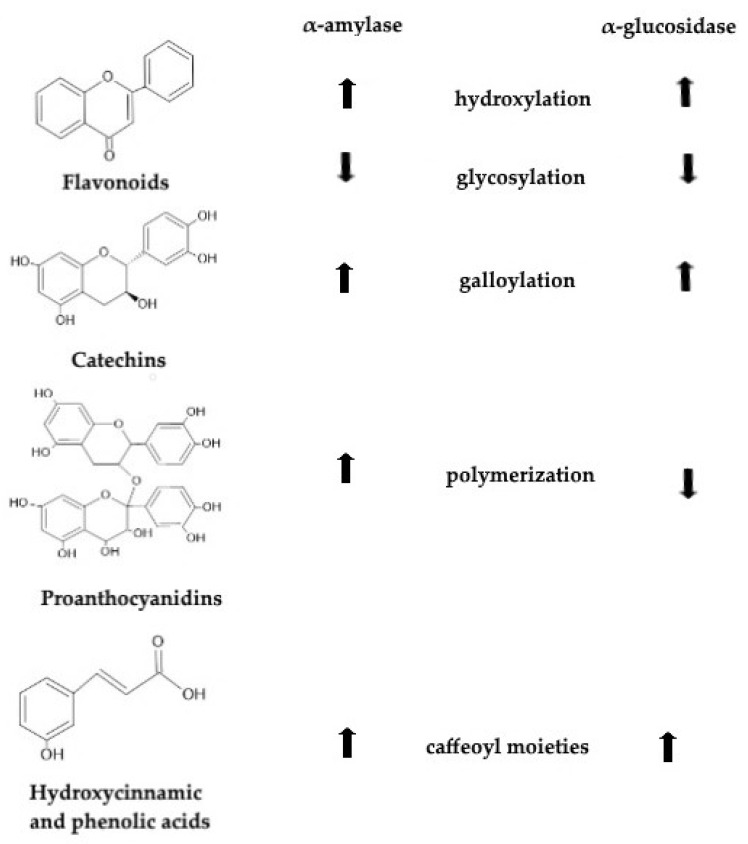
Structure–activity relationship between polyphenols and α-amylase and α-glucosidase enzymes.

To sum up, Figure 1 presents how transformations in the chemical structure of polyphenols affect their activity against α-amylase and α-glucosidase enzymes. These modifications in the chemical structures of certain groups of polyphenols can cause increases or decreases in their inhibitory activity against the mentioned enzymes. Figure 1 presents a quick guide of these changes.

## 8. Conclusions and Future Perspectives

In the present study, structures of polyphenols were evaluated for how they can affect their inhibitory activity against α-amylase and α-glucosidase. To summarize, the hydroxylation of flavonoids, the galloylation of catechins and the presence of caffeoyl moieties improved the activity against both enzymes. Glycosylation of flavonoids, on the other side, caused a decrease in inhibitory activity against α-amylase and α-glucosidase. Polymerization of proanthocyanidins enhanced the inhibitory activity against α-amylase, while it caused decrease in the inhibitory activity against α-glucosidase. Inhibitory activity of dietary polyphenols against α-amylase and α-glucosidase enables retarded starch digestion and the alleviation of postprandial hyperglycemia. Hence, these compounds may be developed as functional foods to prevent and treat type II diabetes. Further research should be focused on the assessing the nature, isolation, purification and analysis of the individual polyphenols that are responsible for the positive effects. Further research should also focus on the potential synergistic effects that polyphenols have with different metabolites. The additional clinical investigations are also required in order to make clear findings concerning the efficacy and safety of both the short-term and long-term administration of polyphenols in people with type II diabetes. There is a need for the development of novel foods enriched with polyphenols and these phytochemicals could be included in a variety of foods, such as bread, baked products or beverages. It is suggested that more research be conducted focusing on the concentrations of polyphenols that should be added to foods that would not affect texture, organoleptic and nutritional properties, as these characteristics are deciding factors in determining acceptability of novel foods on the market.

## Figures and Tables

**Table 1:** Polyphenols from different sources used as inhibitors of α-amylase.

Polyphenols from Plant Material				
Source	Individual Polyphenols	Delivery System	Inhibitory Activity	Ref.
Mulberry fruit (*Morus alba*) extract	Cyanidin-3-glucoside, rutin and gallic acid	Freeze-dried particles prepared from maltodextrin	IC_50_ value for mulberry fruit extract was 0.30 mg/mL and for encapsulated mulberry fruit extract it was 0.28 mg/mL	[62]
Black Caraway (*Nigella sativa*) seeds	n.d.	Extract microencapsulated in β-cyclodextrin	For concentrations of extracts from 10 µg/mL to 160 µg/mL, inhibitory activity (%) ranged from 16.9% to 32.31%	[63]
Minnieroot (*Ruellia tuberosa*) root	n.d.	Chitosan microcapsules crosslinked using sodium tripolyphosphate by spray drying	IC_50_ value of acarbose was 4.81 μg/mL; *R. tuberosa* extract had IC_50_ value of 47.15 μg/mL; microcapsules of *R. tuberosa* extract prepared in pH 4, 0.1% (*w*/*v*) chitosan and 90 min stirring time had IC_50_ value of 50.65 μg/mL	[64]
Cagaita (*Eugenia dysenterica*)	Quercetin and its derivates	Spray-dried and freeze-dried powders produced with gum arabic or inulin	The IC_50_ values were in the range from 10.6 mg/mL to 107 mg/mL for powders prepared with gum arabic and from 9.8 mg/mL to 99.5 mg/mL for powders prepared with inulin	[65]
Cinnamon (*Cinnamomum zeylanicum*) extract	Proanthocyanidins	Spray-dried and freeze-dried microparticles formed using gelatin and five different polysaccharides—pectin, gum arabic, cashew gum, κ-carrageenan and carboxymethylcellulose	Inhibition of α-amylase, expressed as IC_50_ values ranged from 2.6 μg/mL (spray-dried samples) to 4.1 μg/mL (cinnamon extract)	[66]
King’s salad (*Cosmos caudatus*) extracts	n.d.	Spray-dried microcapsules	Microcapsules prepared in pH 4, 0.05% of chitosan and 90 min stirring time had optimum efficiency, with the IC_50_ value of 92.85 μg/mL, pure extract had IC_50_ value of 73.07 μg/mL, while for acarbose it was 4.83 μg/mL	[67]
Stevia (*Stevia rebaudiana*) leaves extract	n.d.	Maltodextrin microcapsules prepared by spray-drying process	For prepared samples, enzyme assay showed lack of inhibition at concentrations from 0.1 to 0.5 mg/mL	[68]
Winemaking byproducts	Malvidin-3-glucoside, quercetin-3-O-glucoside, catechin and epicatechin	Spray-dried grape skin powder and maltodextrin-encapsulated grape skin phenolics	IC_50_ values for grape skin and encapsulated grape skin phenolics were 0.44 mg/mL dry weight and 0.20 mg/mL dry weight	[69]
Blackberry (*Rubus* subg. *Rubus*) juice and apple fiber polyphenols	Cyanidin-3-glucoside, cyanidin 3-dioxalylglucoside, quercetin, ellagic acid, chlorogenic acid, phloretin and phlorizin	Freeze-dried apple fiber/blackberry juice complexes	Inhibition ranged from 22.10% (10% apple fiber addition) to 37.53% (4% apple fiber addition)	[70]
Blue pea *(Clitoria ternatea)* petal flower extract	n.d.	Beads prepared by microencapsulation process	Inhibition (%) of α-amylase was higher for microparticles (22.01%) than for phenolic extract (15.12%)	[71]
Raspberry (*Rubus idaeus*) juice polyphenols	n.d.	Cellulose-based complexes prepared by freeze-drying	Complexes formulated with lower amounts of cellulose had higher inhibition activities (%), for complexes prepared by 15 min of complexation the highest inhibition activity (56.07%) was observed for sample prepared with 2.5% of cellulose	[72]
Black beans (*Glycine max*)	n.d.	Freeze-dried powder prepared using combination of inulin, chitosan, whey protein isolate	Inhibition activity against α-amylase was around 68%	[73]
Olive pomace	Hydroxytyrosol, hydroxytyrosol glucoside, tyrosol and dihydrocaffeic acid	The lyophilisates were prepared using tyrosol in the gelatinized potato starch by mixing conventional and microwave heating during the freeze-drying	Tyrosol lyophilisate under microwave heating had the highest inhibition activity (IC_50_ was 559.57 µg/mL)	[74]
Bitter melon (*Momordica charantia*) juice polyphenols	n.d.	Fresh bitter juice was encapsulated using spray drying, as wall materials maltodextrin, gum arabic, citrus pectin and soy protein isolate were used	The lowest inhibitory activity was observed for encapsulates prepared with pectin and soy protein isolate	[75]
Blackcurrant (*Ribes nigrum*) concentrate	n.d.	Whey protein isolate was used as wall material in spray-drying and freeze-drying techniques	Freeze-dried samples (IC_50_ = 73.46 µg/mL) showed higher inhibitory activity than spray-dried samples (IC_50_ = 81.46 µg/mL)	[76]
Propolis extract	Vanillin, eugenol, ferulic acid, vanillic acid, caffeic acid and p-coumaric acid	Microencapsulation of propolis was conducted using gum arabic and chitosan as wall materials	Inhibition activity (IC_50_) was 0.55 mg/mL	[77]
Tart cherry (*Prunus cerasus*) polyphenols	Cyanidin-3-glucosyl-rutinoside, cyanidin-3- rutinoside, chlorogenic acid, coumaric acid, rutin, quercetin and (-)-epicatechin	Carboxymethylcellulose hydrogels	Inhibition ranged from 21.97% (for hydrogel prepared with 5% carboxymethylcellulose) to around 26% (for hydrogels prepared with 3% and 4% carboxymethylcellulose)	[78]
**Individual Polyphenols**				
**Polyphenol**		**Delivery System**	**Inhibitory Activity**	**Ref.**
Curcumin		Chitosan–alginate polyelectrolyte complex	α-amylase activity of curcumin encapsulated in chitosan–tripolyphosphate was significantly higher after 30 min of incubation, when compared to curcumin nanoencapsulated in chitosan-alginate complex	[79]
Rutin		Rutin was encapsulated with three types of carrier materials (starch, egg albumin, lipid) using three different techniques to investigate the impact of gastrointestinal digestion	Dialysable fractions showed lower anti-diabetic activity than undigested samples (except for the rutin encapsulated in egg albumin which showed the higher inhibition activity than undigested sample)	[80]

n.d.: not defined. IC_50_: the concentration of inhibitor that exhibited 50% inhibition of enzyme activity. Ref.: reference.

**Table 2:** Polyphenols from different sources used as inhibitors of α-glucosidase.

Polyphenols from Plant Material				
Source	Polyphenols	Delivery System	Inhibitory Activity	Ref.
Mulberry fruit (*Morus alba*) extract	Cyanidin-3-glucoside, rutin, gallic acid	Freeze-dried particles prepared from maltodextrin	IC_50_ for mulberry fruit extract was 0.62 mg/mL and for encapsulated mulberry fruit extract it was 0.57 mg/mL	[62]
Cinnamon (*Cinnamomum zeylanicum*) extract	Proanthocyanidins	Spray-dried and freeze-dried microparticles formed using gelatin and five different polysaccharides—pectin, gum arabic, cashew gum, κ-carrageenan and carboxymethylcellulose	Inhibition of α-glucosidase (IC_50_ values) ranged from 0.7 μg/mL (freeze-dried samples) to 6.3 μg/mL (cinnamon extract)	[66]
Winemaking byproducts	Malvidin-3-glucoside, quercetin-3-O-glucoside, catechin and epicatechin	Spray-dried grape skin powder and maltodextrin-encapsulated grape skin phenolics	IC_50_ value of grape skin was 0.28 mg/mL dry weight and for encapsulated grape skin phenolics it was 0.15 mg/mL dry weight	[69]
Red pepper (*Capsicum annuum*) waste	Gallic acid, protocatechuic acid, epicatechin, chlorogenic acid, vanillic acid, caffeic acid, myricetin, quercetin and rutin	Freeze-dried and spray-dried encapsulates prepared to investigate inhibition before and during simulated gastric and intestinal fluids	Freeze-dried samples had higher potential to inhibit α-glucosidase than spray-dried samples, the simulated intestinal fluid showed the highest inhibition activity (%) for freeze-dried (31.6%) and spray-dried (30.42%) samples	[95]
Black beans (*Glycine max*)	n.d.	Freeze-dried powder prepared using combination of inulin, chitosan and whey-protein isolate	Inhibition activity against α-glucosidase was around 68%	[73]
Blackberry juice (*Rubus* subg. *Rubus*) and apple fiber polyphenols	Cyanidin-3-O-glucoside, cyanidin-3-O-dioxalylglucoside, ellagic acid, chlorogenic acid, rutin and phloridzin	Pectin/blackberry hydrogels enriched with apple fiber	IC_50_ value for acarbose was 105.61 µg GAE/mL; for prepared samples IC_50_ values ranged from 1.98 µg GAE/mL (hydrogel with low-methoxylated pectin) to 6.55 µg GAE/mL (hydrogel with high-methoxylated pectin enriched with apple fiber)	[96]
Tart cherry (*Prunus cerasus*) polyphenols	Cyanidin-3-glucosyl-rutinoside, cyanidin-3- rutinoside, chlorogenic acid, coumaric acid, rutin, quercetin and (-)-epicatechin	Carboxymethylcellulose hydrogels	Inhibition ranged from 38.39% (for hydrogel prepared with 5% carboxymethylcellulose) to 56.64% (for hydrogel prepared with 3% carboxymethylcellulose)	[78]
**Individual Polyphenols**				
**Polyphenol**		**Delivery System**	**Inhibitory Activity**	**Ref.**
Catechin hydrate		Starch-based nanoparticles from three sources: horse chestnut, water chestnut and lotus stem	Before the process of simulated gastrointestinal conditions, catechin had inhibitory activity of 92.4%, while free catechin after the process of simulated gastrointestinal conditions had inhibitory activity of 88.79%	[97]

n.d.: not defined. IC_50_: the concentration of inhibitor that exhibited 50% inhibition of enzyme activity. Ref.: reference.

## Data Availability

Not applicable.
